# Supplementary material for: TRPV4 Channels Promote Pathological, but Not Physiological, Cardiac Remodeling through the Activation of Calcineurin/NFAT and TRPC6
Source: Int J Mol Sci. 2024 Jan 26;25(3):1541. doi: 10.3390/ijms25031541 (PMC10855372; doi:10.3390/ijms25031541)
Supplement: Supplementary file 1 [file ijms-25-01541-s001.zip › ijms-2738045-supplementary.pdf]

TRPV4 Channels Promote Pathological, but Not Physiological, Cardiac Remodeling through the Activation of Calcineurin/NFAT and TRPC6

SUPPLEMENTAL MATERIAL

[1] Cardiovascular Diseases Research Group, Vall d'Hebron Institut de Recerca (VHIR), Hospital Universitari Vall d'Hebron, 08035 Barcelona, Spain; laia.yanez@vhir.org (L.Y.-B.); marmoyagimenez@gmail.com (M.M.); antonio.rodriguez.sinovas@vhir.org (A.R.-S.); marisol.ruizmeana@vhir.org (M.R.-M.); javier.inserte@vhir.org (J.I.); elisabet.miro@vhir.org (E.M.); nuriarivasgandara@gmail.com (N.R.); ignacio.ferreira@vhir.org (I.F.G.)
[2] Centro de Investigación Biomédica en Red de Enfermedades Cardiovasculares (CIBERCV), Instituto de Salud Carlos III, 28029 Madrid, Spain
[3] Bio-Heart Cardiovascular Diseases Research Group, Bellvitge Biomedical Research Institute (IDIBELL), L'Hospitalet de Llobregat, 08908 Barcelona, Spain; mtajes@idibell.cat
[4] Institute for Biomedical Research August Pi i Sunyer (IDIBAPS), 08036 Barcelona, Spain; mbatlle@clinic.cat (M.B.); eguasch@clinic.cat (E.G.); amasstachurska@psmar.cat (A.M.-S.)
[5] Cardiology Department, Hospital Clínic, 08036 Barcelona, Spain
[6] Cardiology Department, Hospital del Mar, 08003 Barcelona, Spain
[7] Cardiology Department, Hospital Universitari Vall d'Hebron, 08035 Barcelona, Spain
[8] Department of Medicine, Universitat Autònoma de Barcelona, 08193 Barcelona, Spain
[9] Centro de Investigación Biomédica en Red en Epidemiología y Salud Pública (CIBERESP), Instituto de Salud Carlos III, 28029 Madrid, Spain
[10] Department of Clinical Research, ASCIRES-CETIR Biomedic Group, 08029 Barcelona, Spain; anna80@gmail.com
* Correspondence: begona.benito@vhir.org or b.benito.v@gmail.com; Tel.: +34-656192261
† These authors contributed equally to this work.

FIGURES

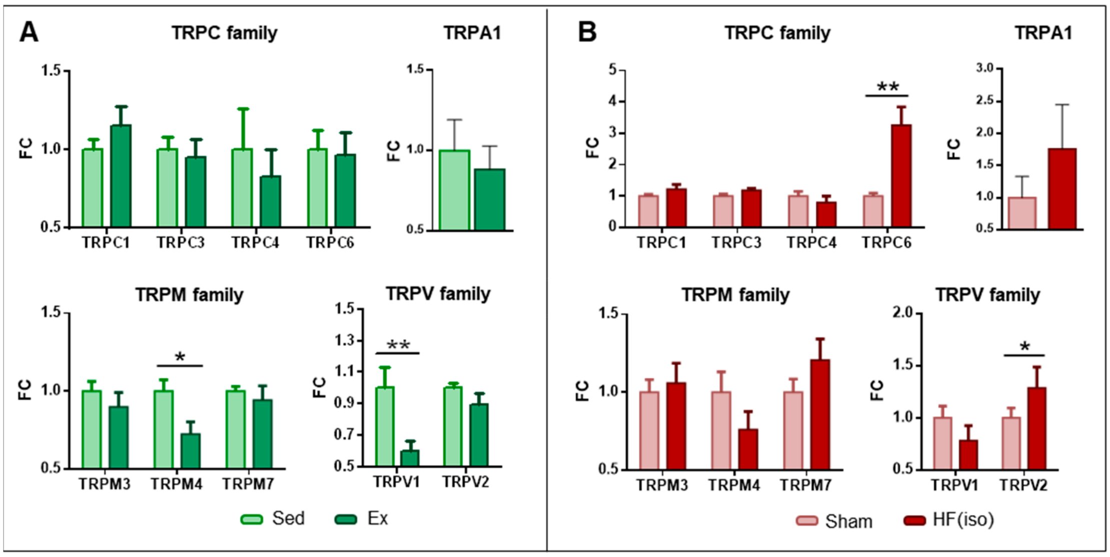

Figure S1: Gene expression levels of cardiac mechanoreceptors in the LV of the 4 study groups generated in mice (n=6-12/group); Ex versus Sed, green bars (Figure S1A); and HF(iso) versus sham, red bars (Figure S1B). FC: fold-change. * p <0.05; ** p <0.01. Differences between groups were examined using t-test.

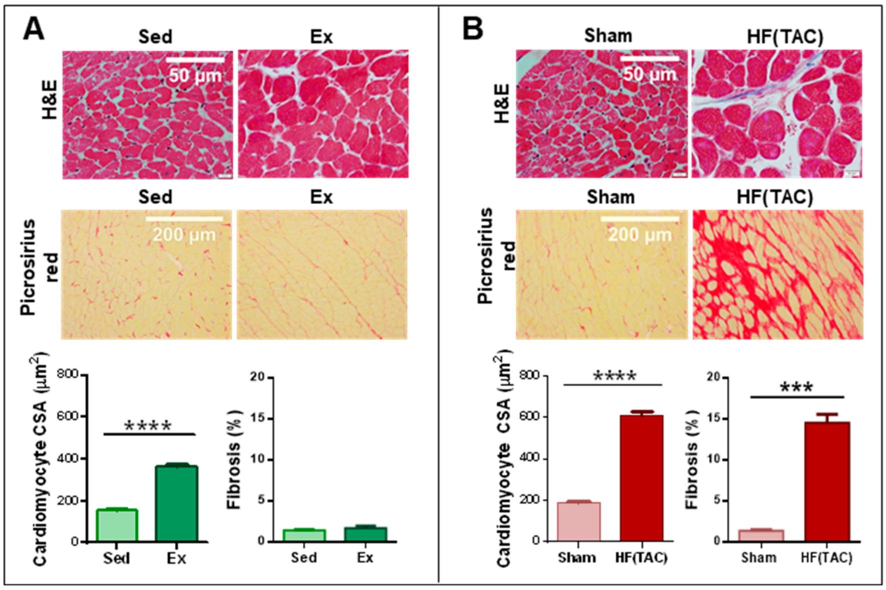

Figure S2: Differential features regarding cardiomyocyte cross-sectional area (CM-CSA) and collagen deposition (% Fibrosis) between physiological and pathological remodeling in rats (n=6-8/group); Representative images of CM-CSA (stained with Hematoxylin & Eosin (H&E), scale bar corresponding to 50 µm) and fibrosis (stained with picrosirius red, scale bar corresponding to 200 µm) are shown at the top and quantifications at the bottom.; Ex versus Sed (Figure S2A), and HF(TAC) versus sham (Figure S2B). CM: cardiomyocyte; CSA: cross-sectional area; *** p <0.001; **** p <0.0001. Differences between groups were examined using t-test.

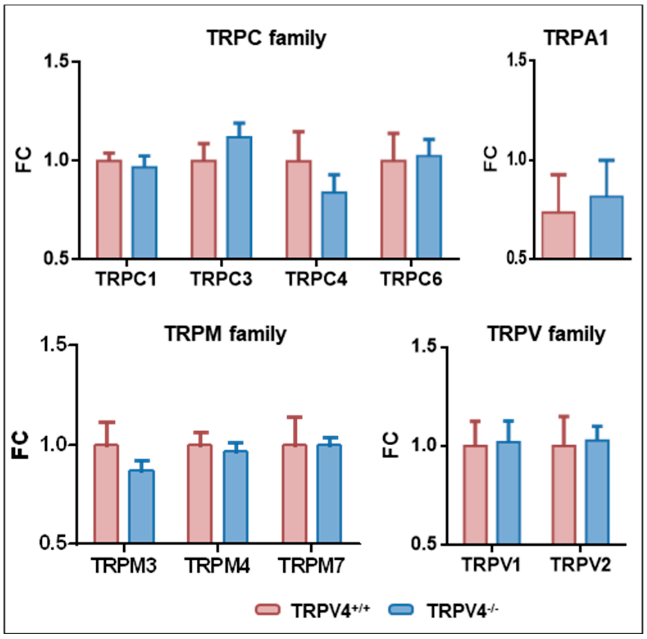

Figure S3: Gene expression of TRP channels in TRPV4+/+ and TRPV4-/- mice at baseline (n=7-8/group). FC: fold-change. Differences between groups were examined using t-test.

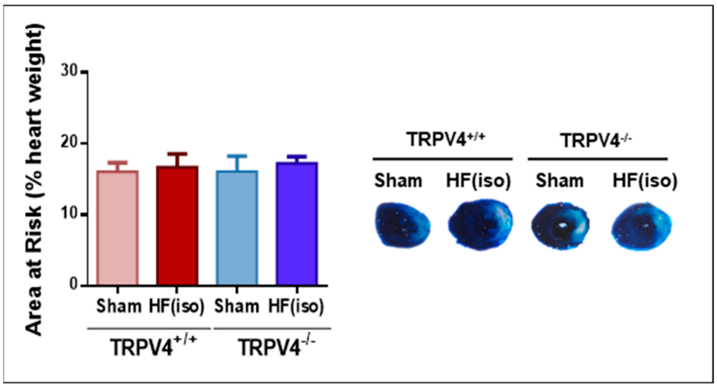

Figure S4: Area at risk (AAR), representing the territory perfused by the ligated anterior descending coronary artery, and exposed by no staining after perfusion with Evans Blue in the four study groups (n=5-8/group). AAR is normalized by the total heart weight. A representative image is shown for each group. Data were analyzed with one-way ANOVA.

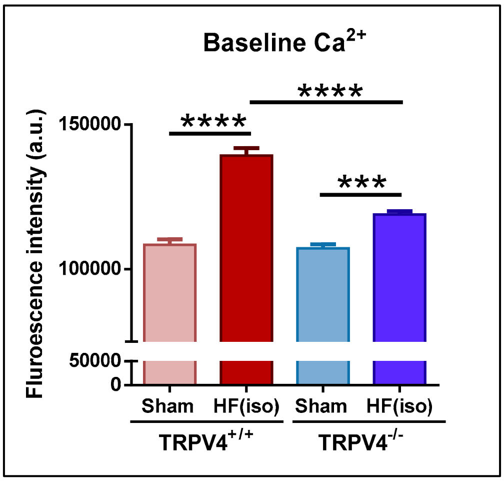

Figure S5: Ca^{2+} levels (Fluorescence intensity, arbitrary units (a.u.)) at baseline in fibroblasts (FB) from all experimental groups.

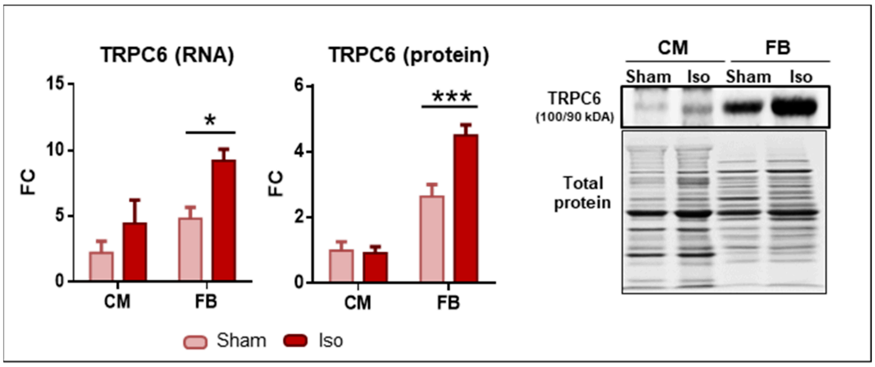

Figure S6: Relative expression of TRPC6 in cardiomyocytes (CM) and fibroblasts (FB) (n=6/group). * $p<0.05$; *** $p<0.001$. Differences between groups were examined with two-way ANOVA.

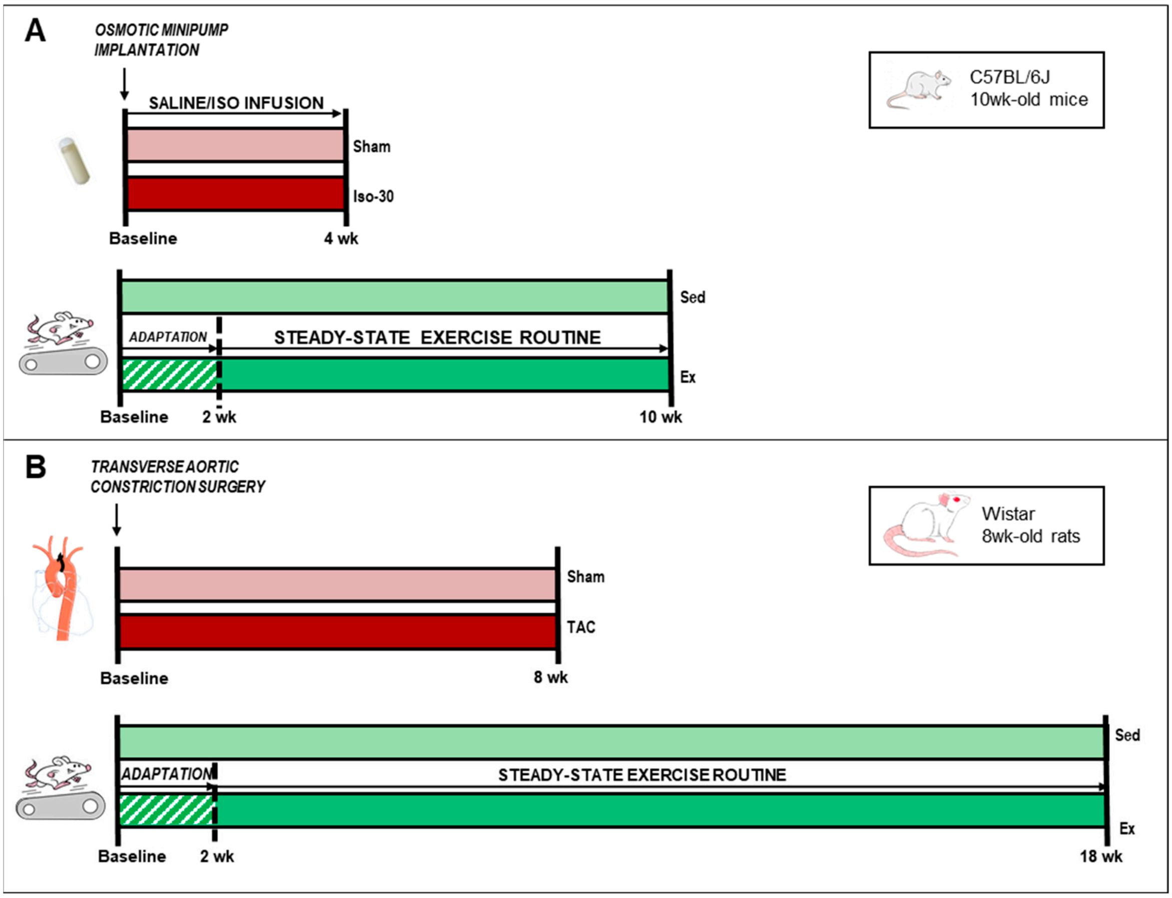

Figure S7: Outline representing the animal models of physiological and pathological cardiac remodeling used in mice (Figure S6A) and rats (Figure S6B).

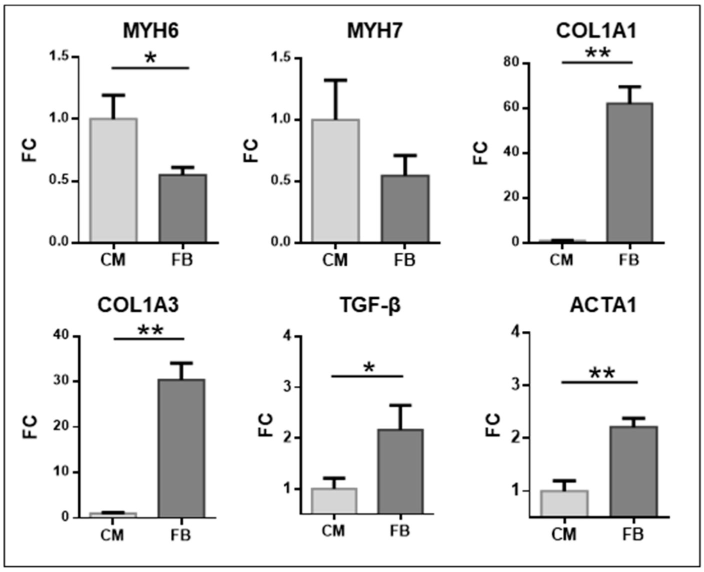

Figure S8: Differential gene expression of acta1, TGFβ1, FN1, Col1a1, col3a1, MYH6 and MYH7 between the CM and non-CM fractions obtained after specific digestion in Langendorff-perfused hearts. The results confirm the presence of CM in the CM fraction and the presence of FB in the non-CM fraction. * $p<0.05$; ** $p<0.01$. Differences between CM and FB were examined with t-test.

Table S1: Specific PCR probes

Gene	Specie	Assay ID
GAPDH	Mouse	Mm99999915_g1
	Rat	Rn01775763_g1
COL1A1	Mouse	Mm00801666_g1
ACTA2	Mouse	Mm01546133_m1
TRPC1	Mouse	Mm00441975_m1
TRPC3	Mouse	Mm00444690_m1
TRPC4	Mouse	Mm00444280_m1
TRPC5	Mouse	Mm00437183_m1
TRPC6	Mouse	Mm01176083_m1
	Rat	Rn00677559_m1
TRPM3	Mouse	Mm01210379_m1
TRPM4	Mouse	Mm00613173_m1
TRPM7	Mouse	Mm00457998_m1
TRPA1	Mouse	Mm01227437_m1
TRPV1	Mouse	Mm01246302_m1
TRPV2	Mouse	Mm00499025_m1
TRPV4	Mouse	Mm00499025_m1
	Rat	Rn07312319_m1

METHODS

Animals

Experiments were performed in 10-week-old C57BL/6J mice (25-30 g) and 8-week-old Wistar rats (200-250 g). Additionally, transgenic C57BL/6J wildtype (WT, TRPV4+/+) and TRPV4 knockout (KO, TRPV4-/-) mice at the age of 10 weeks were used in some experiments. The transgenic TRPV4 model is a constitutive knock-out generated by Cre-Lox-mediated excision of exon 12, which encodes the pore-loop region and the transmembrane adjacent domains of TRPV4. The excision of this exon renders a non-functional TRPV4 polypeptide chain that is targeted for degradation.[1]

In vivo models of adverse and adaptive cardiac remodeling

- *Mouse model of adverse cardiac remodeling induced by chronic infusion of isoproterenol*

Pathological remodeling was generated using a previously validated model of HF induced by 28-day continuous infusion of isoproterenol.[2] Under 2% isoflurane anesthesia, osmotic minipumps (1004 Alzet, Cupertino, CA, USA) were surgically implanted in a subcutaneous pocket created by blunt dissection at the back of the animals. The pumps, with a reservoir of approximately 100 μL and allowing a continuous and passive infusion at 0.11 μL/h, were set to deliver either isoproterenol (30 mg/kg/day, HF(iso) group) or saline (0.9% NaCl, sham group) for 28 days, when cardiac remodeling was assessed.

- *Mouse model of adaptive cardiac remodeling induced by moderate exercise training*

Exercise mice (Ex group) were conditioned to run on a treadmill (LE8710, Panlab) at a moderate intensity, using an adapted protocol from previous publications.[3] The treadmill allowed for different animals to run simultaneously in different lanes and had a grid in the back that would administer a small electric shock (2 mA) on contact to ensure that the animals ran effectively without being harmful.[3] Treadmill speed, slope and training duration were progressively increased during a 2-week adaptation period, after which a stable routine (speed of 15 cm/s, with a 6° positive slope during 30 minutes) was kept and repeated 5 days a week for 8 weeks. All sessions were supervised by an experienced investigator to ensure proper running and no animal stress. Sedentary mice (Sed group) were housed in the same conditions but did not perform any exercise session.

- *Rat model of adverse cardiac remodeling induced by transverse aortic constriction*

Transverse aortic constriction (TAC) surgery was performed in male Wistar rats under general anesthesia and mechanical ventilation, with the help of a surgical lens. After medial suprasternal partial thoracotomy, the aortic arch was exposed. A bended and blunted 20G needle was placed between the brachiocephalic trunk and the left carotid artery, and a 5.0 nylon suture was tied around both the vessel and the needle to restrict blood flow. The needle was subsequently removed in order to yield an aortic constriction of 0.9 mm in diameter, as previously described.[4] Littermate sham controls underwent the same surgery, but no constriction was applied (sham group). After both TAC and sham surgery, the chest cavity and the skin were carefully sutured. After surgery, rats were placed back to their cages and monitored regularly to ensure complete recovery. The endpoint was established 8 weeks after surgery, when pathological remodeling should already be developed according to previous reports.[4]

Rat model of physiological cardiac remodeling induced by moderate exercise training

Exercise rats (Ex group) underwent daily running training sessions on a treadmill (LE8710, Panlab). The protocol included a progressive training program, starting with a 10-minute running session at 10 cm/s, followed by gradual increase of both speed and duration until a steady-state routine (speed 35 of cm/s, 0º slope during 45 minutes) was achieved after 2 weeks. Thereafter, animals were trained at this intensity 5 days a week for 16 weeks.[3] Sedentary rats (Sed group) were housed and fed in the same conditions without performing any exercise.

Schematic representation of the animal models of physiological and pathological remodeling is depicted in Figure S7. In all models, at the final endpoint, animals were euthanized by intraperitoneal (IP) injection of sodium pentobarbital (100 mg/kg). The hearts were removed and either cannulated on a Langendorff perfusion system, weighted and fixed for histology, or snap-frozen in liquid nitrogen. All stored samples were kept at -80 ºC.

Echocardiography measurements

Echocardiographic acquisitions were taken using a Vivid IQ system and a L8-18 Linear Array 5-15 MHz probe (General Electric Healthcare, Horten, Norway). Animals were anesthetized with 2% isoflurane, and hearts were imaged in parasternal short-axis projections. M-mode echocardiograms of the mid-ventricle were recorded at the level of papillary muscles and used to measure the left ventricular diameter at the end of the diastole (LVDd) and the intraventricular septum thickness (IVS). The ejection fraction (EF) was calculated by the formula packed in GE Healthcare Ultrasound Vivid7 system and proposed by the American Society of Echocardiography. The average of 3 consecutive cardiac cycles was used for each measurement. All measures were taken blinded.

Histology

Heart samples fixed in buffered 4% formaldehyde were embedded in paraffin and cut into 4-µm thick slices. LV cardiomyocyte cross-sectional area (CM CSA) was measured in transverse sections stained with haematoxylin and eosin (H&E). Random phomicrographs of each heart section were taken at 40X and CM CSA was measured by outlining round to cuboidal-shaped nucleated cells. At least 25 random cells from each slide were measured using the Image J software (Image J, U.S. National Institutes of Health, USA). LV fibrosis was assessed by staining heart sections with Picrosirius red. Ten representative photomicrographs per animal were acquired at 40X and used to quantify collagen deposition using Image J software. Perivascular, pericardial and endocardial collagen were excluded from measurements. All measures were taken and analyzed blinded.

Langendorff-perfused hearts

Immediately after sacrifice, the hearts were cannulated via the aorta into a standard Langendorff perfusion system, secured with a 3-0 suture, and kept beating with a retrograde perfusion through the aorta at a constant flow with an oxygenated (95% O2: 5% CO2) Krebs solution at 37 ºC (118 mM NaCl, 4.7 mM KCl, 1.2 mM MgSO4, 1.8 mM CaCl2, 25 mM

NaHCO3, 1.2 mM KH2PO4, 11 mM glucose, pH 7.4). The setup was adjusted to produce a perfusion pressure of 80-90 mmHg (normoxic environment).

Assessment of arrhythmias

- *Electrophysiological studies*

Arrhythmia inducibility was assessed in Langendorff-perfused hearts. Arrhythmogenesis was monitored by bipolar electrogram recordings using stainless steel electrodes (6491 unipolar pediatric temporary pacing lead, Medtronic) placed both at the LV base and the aortic cannula. Signals were amplified, digitized at 2 kHz, and stored for later analysis using PowerLab/8SP data acquisition System and Chart 5.0 (AD Instruments). The electrophysiological study protocols were initiated after 20 mins of stabilization. First, spontaneous arrhythmias were examined during 5 minutes. Then, arrhythmia inducibility was assessed by ventricular stimulation. Electrical stimulation was achieved using paired stainless- steel electrodes (6491 unipolar pediatric temporary pacing lead, Medtronic) placed at the apex of the LV. Regular pacing was set at 2V using rectangular pulses of 1ms duration. A modified version of a validated stimulation protocol was used to induce ventricular tachyarrhythmias (VTA).[5] Ventricular effective refractory period was calculated by applying a premature stimulus (S2) at the pacing site after a train of 18 stimuli (S1). The effective refractory period was defined as the longest S1-S2 interval that produced a propagated response. VTA were induced using a protocol of programmed electrical stimulation consisting of repetitive trains of 18 stimuli (S1) delivered at a basal cycle length (BCL) of 100 ms, followed by a premature extra stimulus (S2) introduced by 2 ms decrements, starting at 60 ms or at an interval 5 ms longer than the effective refractory period until a minimal coupling interval of 30 ms. Subsequently, a second and third extrastimuli were progressively introduced (S3-S4) following the same principle. Arrhythmia induction was further assessed after burst pacing at rates of 50, 40 and 30 ms during 5 s, each cycle repeated three consecutive times. Induced arrhythmias were recorded if occurred within the first 3 s after the last stimulus. The same stimulation protocol was repeated after regional ischemia.

- *Induction of regional ischemia*

Regional ischemia was induced in Langendorff-perfused hearts by ligation of the left descending coronary artery using a 6.0 silk snare placed 2-3 mm distal to its origin. Successful coronary occlusion was verified by assessing changes in the ECG (ST-segment elevation) and an increase in LV perfusion pressures. Spontaneous arrhythmias were analyzed for a 5-minute period starting 15 min after the ischemia, and inducibility of VTA was tested thereafter.

At the end of the protocol, the size of the ischemic area, or area at risk (AAR), was measured in all hearts. The hearts were perfused with 0.5 mL of 5% Evans Blue diluted in saline, which stained the entire myocardium, except for the area irrigated by the ligated artery. The non-stained area was considered the AAR. Stained hearts were cut into 4 transversal slices and imaged. Digitized images were measured semi-automatically with the software Image Pro-Plus (Media Cybernetics). The AAR and the total area (TA) of each slice were normalized to the heart's weight. The final AAR and TA were calculated summing up the values of all 4 slices as follows:[6] $AAR/TA\ (\%) = (\Sigma\ AAR1\text{-}4 / \Sigma\ TA1\text{-}4) * 100$.

Isolation of ventricular cardiomyocytes and fibroblasts

Cardiomyocytes (CM) and fibroblasts (FB) were isolated from hearts hung in a Langendorff system retrogradely perfused with warm oxygenated Krebs solution and then enzymatically digested with 0.4 mg/mL type 2 collagenase (Worthington Biochemical Corporation, Lakewood, NJ, USA) during 20 minutes. Further digestion was achieved by mincing the LV into small pieces followed by pipetting. The resulting cell suspension was filtered through a 180 µm pore size mesh, and CM were pelleted by centrifugation at 25 g. Supernatants were meshed again through a 40 µm cell strainer (Thermofisher, Waltham, MA, USA) and the non-myocyte fraction (containing FB) was pelleted by centrifugation at 650 g. The presence of FB in the non-myocyte fraction and the presence of CM in the myocyte fraction were confirmed by the detection of characteristic FB genes (Col1a1, col3a1, TGFβ1, acta1) with no significant MYH6 or MYH7 expression in the former and expression of MYH6 and MYH7 in the latter (Figure S8).

Fibroblast cultures

Freshly-isolated FB were cultured in DMEM medium (#30-2002; ATCC, Manassas, VA, USA) supplemented with 10% FBS and 2% penicillin/streptomycin for 2 hours at 37 ºC and 5% CO2, and washed afterwards. Only viable FB, attached on the plate, were kept, while other cells were washed out. The medium was changed after 24 hours, and fibroblasts were allowed to grow until confluence.

qPCR analyses

qPCR analyses were performed in LV samples, freshly-isolated FB or cultured FB by extracting RNA with the Nucleospin RNA extraction kit (Macherey-Nagel, Düren, Germany), which was then retrotranscribed into cDNA with a High-Capacity cDNA Reverse Transcription Kit (Applied Biosystems, Waltham, MA, USA). In a set of experiments (Figure 6G), aimed to study the consequences of TRPV4 activation on TRPV4 and TRPC6 expression, FB from WT animals were treated for 4h with GSK1016790A (GSK, 100 nM) either in the absence or presence of the calcineurin inhibitor cyclosporine A (CsA, 1µM, 1h before GSK challenge) before total RNA was extracted. Gene expression was measured in triplicate in a 7900HT Fast Real-Time PCR System (Applied Biosystems, Waltham, MA, USA) using TaqMan Universal PCR master mix (Thermofisher, Waltham, MA, USA) and pre-designed gene-specific probes (Thermofisher, Waltham, MA, USA, see Supplementary Table S1). Relative quantification was calculated using the comparative threshold method and expressed as fold change (FC) over control group.

Western blotting

Total protein from tissues and cells was extracted with RIPA buffer (50 mM Tris Base, 150 mM NaCl, 10 mM EDTA, 0.1 % SDS, 0.5 % Na-deocycholate, 1% Triton-X-100, 10 mM NaF, 2 mM Na3VO4, 1 % protease inhibitor, pH 7.3). Protein samples were separated in a 10% SDS-PAGE gel electrophoresis, transferred to nitrocellulose membranes and blocked with 5% non-fat milk TTBS. Membranes were probed overnight at 4 ºC with the following antibodies: TRPV4 (ACC034, 1:500; Alomone labs, Jerusalem, Israel), TRPC6 (PA5-77308,

1:500 or PA5-29848, 1:1000; Thermofisher, Waltham, MA, USA), CaN (610259, 1:500; BD Biosciences, Franklin Lakes, NJ, USA) and GAPDH (GT239, 1:10000; GeneTex, Irvine, CA, USA) as the endogenous control. Antibody and isoform specificity were validated using the KO model for TRPV4. However, due to the unavailability of a TRPC6 KO control, direct assessment of isoform specificity for TRPC6 was not feasible. To address this limitation, the samples were incubated with a blocking peptide. After three washes, membranes were incubated with peroxidase-conjugated secondary anti-rabbit or mouse IgG for 1 hour at room temperature. After washings, proteins were developed with ECL (Amersham Biosciences, Amersham, UK) and captured using an Odyssey FC Imaging System (LI·COR Biosciences, Licoln, NE, USA). Band intensities were measured by densitometry scanning with Image Studio Lite software.

Fluorescence Ca2+ imaging

Passage-1 FB were seeded at a density of 2x105 cells/well on 96 well-plates and allowed to grow for 24 hours. The calcium sensitive dye Fluo-4 AM (5 μM) was used to measure changes in the concentration of intracellular calcium. Fluorescence was continuously monitored at an excitation wavelength of 488 nm and emission of 528 nm with the microplate reader SpectraMax ID3 (Molecular Devices). To load the cells with the dye, cells were rinsed with an isotonic solution (~300 mOsm) containing: 139 mM NaCl, 3.6 mM KCl, 1 mM CaCl2, 1.2 mM MgSO4, 10 mM HEPES, 5mM glucose, pH 7.4. Then, FB were loaded with Fluo-4 AM for 30 minutes at 37 ºC. Following incubation, cells were washed with isotonic solution for 30 minutes at 37 ºC.

Basal intracellular calcium concentration was first recorded, measuring the fluorescence every 15 seconds during 90 seconds (basal fluorescence, F0). After that, cells were treated either with one of the following TRPV4 activators: GSK1016790A (GSK10, 100 nM) or an hypoosmotic solution (~140 mOsm) containing: 50 mM NaCl, 3.6 mM KCl, 1 mM CaCl2, 1.2 mM MgSO4, 10 mM HEPES, 5 mM glucose, pH 7.4. Measurements were continued every 3 seconds up to 3 minutes. To enhance changes in cytosolic calcium, the calcium reuptake to the endoplasmic reticulum was inhibited by adding 1 μM of thapsigargin, the sarco/endoplasmic calcium ATPase (SERCA) inhibitor. In some experiments FB were preincubated with 10 μM HC-067047 (HC), the TRPV4 antagonist, during 3 minutes before introducing GSK or the hypoosmotic solution. Both GSK and HC were dissolved in DMSO at a 10 mM stock concentration and were freshly diluted into their working solution just before use. Changes in intracellular calcium were calculated as the ratio of Fluo-4 fluorescence intensity at each time point relative to the basal fluorescence (F/F0).

Calcineurin activity assay

Calcineurin enzymatic activity was measured in protein extracts collected from isolated non-myocyte fractions using the calcineurin cellular activity assay kit (BML-AK816-0001; Enzo Life Sciences, Farmingdale, NY, USA) following manufacturer's instructions. Briefly, non-myocyte fractions were lysed with lysis buffer and mechanically dissociated with a 27 G needle. Samples were centrifuged at maximum speed for 10 minutes at 4 ºC. Supernatants were collected and desalted using a desalting column resin. Calcineurin activity was quantified by detection of free phosphate released by measuring the absorbance of malachite green (OD 630 nm).

NFAT nuclear translocation

FB at passage-1 were seeded in 8-chamber culture slides (2 x 10^4 cells/well) coated with 0.02% gelatin, 5 μg/ml fibronectin and allowed to grow for 24 hours. Then, cells were fixed in 4 % formol, permeabilized with 0.2 % tween-20 for 5 minutes and blocked with 5 % rabbit serum. Primary antibodies (NFATc3 1:100; ab219063, Abcam, Cambridge, UK) were incubated overnight at 4 ºC. After three wash-outs, cells were incubated with secondary anti-mouse antibody conjugated to an Alexa Fluor 546 for 1 hour, and nuclei were stained with Hoechst (5 μg/ml).

In a first set of experiments (Figure 5), the positive control was performed by inducing calcineurin activation by increasing the extracellular calcium concentration (4 mM) during 2 hours before fixation. Results were compared to those obtained TRPV4+/+ and TRPV4-/- mice subjected to isoproterenol infusion or sham. In a second set of experiments (Figure 6), NFAT translocation was analyzed in different experimental conditions: 1) TRPV4 activation by GSK10 (100 nM, for 4h; 2) TRPC6 activation by GSK1702934A (GSK17, 1 μM) For 4h; 3) simultaneous activation of TRPV4 and TRPC6 (GSK10 100 nM + GSK17 1 μM); 4) GSK10 in the presence of the TRPC6 inhibitor BI-749327 (BI, 1 μM), administered 1h before the activator; 5) GSK17 (1 μM) in the presence of the TRPV4 inhibitor HC (10 μM), administered 1h before the activator; and 6) control (no activators nor inhibitors present).

In all cases, images were acquired at 400X magnification using an Olympus FV1000 fluorescence microscope and analysed using Image J. NFAT translocation was measured as the ratio of mean fluorescence intensity at the nucleus relative to that at the cytosol. At least 30 isolated FB were measured per condition in 4 independent experiments.

Statistical analysis

Data are presented as mean ± standard error of the mean (SEM). For variables following a Gaussian distribution, statistical analyses were performed using t-test (for comparisons between 2 groups), or one way (for comparisons between 3 or more groups). If data did not respect normality, non-parametric tests were used. A two-way ANOVA was used to assess differences in the mRNA levels between CM and FB, where factors were cell type, and study group. A two- way ANOVA was also used in most of the experiments performed in transgenic mice to evaluate the effects of TRPV4 deletion, where factors were genotypes (TRPV4+/+ and TRPV4-/-) and treatment group (sham and iso). Since arrhythmia inducibility did not follow a Gaussian distribution, differences during normoxia and after ischemia were assessed by a non-parametric equivalent of a two-way ANOVA test (Scheiner-Ray-Hare, SRH test). All ANOVA and non- parametric analyses were followed by a Bonferroni post hoc correction when interaction was found. Data were analyzed using GraphPad Prism 6.0 and differences were considered statistically significant when $p < 0.05$.

Study approval

All experimental protocols were approved by the institutional Animal Research Ethics Committees (Vall d'Hebron Research Institute, Hospital del Mar Medical Research Institute, Institut d'Investigació August Pi I Sunyer) and the Generalitat de Catalunya (CEAOH/9538_MR1/1).

REFERENCES

1. Liedtke, W.; Friedman, J.M. Abnormal Osmotic Regulation in Trpv4-/- Mice. **2003**, *100*, 13698–13703.
2. Yáñez-Bisbe, L.; Garcia-Elias, A.; Tajes, M.; Almendros, I.; Rodríguez-Sinovas, A.; Inserte, J.; Ruiz-Meana, M.; Farré, R.; Farré, N.; Benito, B. Aging Impairs Reverse Remodeling and Recovery of Ventricular Function after Isoproterenol-Induced Cardiomyopathy. *International journal of molecular sciences* **2021**, *23*, doi:10.3390/IJMS23010174.
3. Benito, B.; Gay-Jordi, G.; Serrano-Mollar, A.; Guasch, E.; Shi, Y.; Tardif, J.-C.; Brugada, J.; Nattel, S.; Mont, L. Cardiac Arrhythmogenic Remodeling in a Rat Model of Long-Term Intensive Exercise Training. *Circulation* **2011**, *123*, doi:10.1161/CIRCULATIONAHA.110.938282.
4. Batlle, M.; Castillo, N.; Alcarraz, A.; Sarvari, S.; Sangüesa, G.; Cristóbal, H.; De Frutos, P.G.; Sitges, M.; Mont, L.; Guasch, E. Axl Expression Is Increased in Early Stages of Left Ventricular Remodeling in an Animal Model with Pressure-Overload. *PLoS ONE* **2019**, *14*, doi:10.1371/journal.pone.0217926.
5. Sanchez, J.A.; Rodriguez-Sinovas, A.; Fernandez-Sanz, C.; Ruiz-Meana, M.; Garcia-Dorado, D. Effects of a Reduction in the Number of Gap Junction Channels or in Their Conductance on Ischemia-Reperfusion Arrhythmias in Isolated Mouse Hearts. *American journal of physiology. Heart and circulatory physiology* **2011**, *301*, H2442-53, doi:10.1152/ajpheart.00540.2011.
6. Fernández-Friera, L.; García-Ruiz, J.M.; García-Álvarez, A.; Fernández-Jiménez, R.; Sánchez-González, J.; Rossello, X.; Gómez-Talavera, S.; López-Martín, G.J.; Pizarro, G.; Fuster, V.; et al. Accuracy of Area at Risk Quantification by Cardiac Magnetic Resonance According to the Myocardial Infarction Territory. *Revista espanola de cardiologia (English ed.)* **2017**, *70*, 323–330, doi:10.1016/j.rec.2016.07.004.
